# EVALUATION OF A CAREGIVER FINANCIAL PREPAREDNESS PROGRAM: SIGNIFICANT SHORT- AND LONG-TERM IMPACTS

**DOI:** 10.1093/geroni/igac059.1148

**Published:** 2022-12-20

**Authors:** Katherine Judge, Claire Grant, Monica Moreno, Kerry Lanigan, Lauren Stratton, Sam Fazio

**Affiliations:** Cleveland State University, Cleveland, Ohio, United States; Cleveland State University, Cleveland Heights, Ohio, United States; Alzheimer's Association, Chicago, Illinois, United States; Alzheimer's Association, Chicago, Illinois, United States; Alzheimer's Association, Chicago, Illinois, United States; Alzheimer's Association, Chicago, Illinois, United States

## Abstract

Over 50 million informal caregivers provide care to an older adult age 50 or older. Negative financial impacts include significant out-of-pocket expenses, decreased income and future earnings. The program, Managing Money: A Caregiver’s Guide to Finances, was developed to address the lack of evidence-based programs and evaluated using a randomized control trial across 5 time points: Time 1 (baseline); Time 2 (after condition completion); Time 3 (30 days after Time 2); Time 4 (60 days after Time 2); and Time 5 (90 days after Time 2). The mean age of participants was 55.45 (SD=14.85); 80% female; 72% White and 69% married. Forty-six percent provided care to their parent/in-law, 36% to a spouse/partner, and 40% were employed full-time. Participants were randomly assigned to the program (N=76) or waitlist condition (N=98). No significant differences on demographic or care context variables were found between conditions. A 2 x 5 repeated measures analyses examined significant change across time based on condition. For significant outcomes, post-hoc analyses examined whether change was short-term (T2,T3); long-term (T4,T5); or both. Results found: 1) significant short- and long-term improvements for Unmet Needs (F(1,4)=8.34,p=.01); Self-efficacy (F(1,4)=4.27,p=.01); and Behavioral Intentions (F(1,4)=3.63,p=.01) and 2) significant long-term improvements for Unmet Needs Distress (F(1,4)=5.82,p=.01) and Behavioral Actions (F(1,4)=4.55,p=.01). Results indicated the program was efficacious in positively impacting key financial preparedness measures. Discussion highlights key study elements including program accessibility and scalability; generalizability of findings and limitations; and contextualizing results within the larger literature.

